# Anticoagulant Activity of Low-Molecular Weight Compounds from *Heterometrus laoticus* Scorpion Venom

**DOI:** 10.3390/toxins9110343

**Published:** 2017-10-26

**Authors:** Thien Vu Tran, Anh Ngoc Hoang, Trang Thuy Thi Nguyen, Trung Van Phung, Khoa Cuu Nguyen, Alexey V. Osipov, Igor A. Ivanov, Victor I. Tsetlin, Yuri N. Utkin

**Affiliations:** 1 Institute of Applied Materials Science, Vietnam Academy of Science and Technology, Ho Chi Minh City 700000, Vietnam; vuthien82@yahoo.com (T.V.T.); hnanh52@yahoo.com (A.N.H.); nckhoavnn@yahoo.com (K.C.N.); 2 Vietnam Academy of Science and Technology, Graduate University of Science and Technology, Ho Chi Minh City 700000, Vietnam; 3 Faculty of Pharmacy, Nguyen Tat Thanh University, Ho Chi Minh City 700000, Vietnam; thuytrangd07@yahoo.com; 4 Istitute of Chemical Technology, Vietnam Academy of Science and Technology, Ho Chi Minh City 700000, Vietnam; trung_cnhh@yahoo.com; 5 Shemyakin-Ovchinnikov Institute of Bioorganic Chemistry, Russian Academy of Sciences, Moscow 117997, Russia; osipov-av@ya.ru (A.V.O.); chai.mail0@gmail.com (I.A.I.); victortsetlin3f@gmail.com (V.I.T.)

**Keywords:** venom, scorpion, blood coagulation, adenosine, peptide

## Abstract

Scorpion venoms are complex polypeptide mixtures, the ion channel blockers and antimicrobial peptides being the best studied components. The coagulopathic properties of scorpion venoms are poorly studied and the data about substances exhibiting these properties are very limited. During research on the *Heterometrus laoticus* scorpion venom, we have isolated low-molecular compounds with anticoagulant activity. Determination of their structure has shown that one of them is adenosine, and two others are dipeptides LeuTrp and IleTrp. The anticoagulant properties of adenosine, an inhibitor of platelet aggregation, are well known, but its presence in scorpion venom is shown for the first time. The dipeptides did not influence the coagulation time in standard plasma coagulation tests. However, similarly to adenosine, both peptides strongly prolonged the bleeding time from mouse tail and in vitro clot formation in whole blood. The dipeptides inhibited the secondary phase in platelet aggregation induced by ADP, and IleTrp decreased an initial rate of platelet aggregation induced by collagen. This suggests that their anticoagulant effects may be realized through the deterioration of platelet function. The ability of short peptides from venom to slow down blood coagulation and their presence in scorpion venom are established for the first time. Further studies are needed to elucidate the precise molecular mechanism of dipeptide anticoagulant activity.

## 1. Introduction

Scorpions (order Scorpiones) are distributed mainly in the hot areas and in the warmer regions of the temperate zone. Scorpion venoms are complex mixtures of compounds represented mainly by peptides and proteins. They manifest mostly neurotoxic effects and instantly paralyze small prey. A sting of large tropical scorpions can be fatal to humans, the main symptom being nervous system damage.

Forest scorpion *Heterometrus laoticus* (family Scorpionidae) occupies the Indochinese peninsula and can be often found in South-West Vietnam [1]. Among the symptoms of *H. laoticus* envenomation are local pain, inflammation, edema, swelling and redness of the stung area, lasting from a few hours to a few days [2]; no human fatalities have been reported so far. *H. laoticus* venom showed both anti-nociceptive and anti-inflammatory activity at subcutaneous injection [3]. A few toxins were isolated from this venom and characterized. The toxin heteroscorpine-1 [4] inhibited growth of bacteria and showed high homology to polypeptide toxins from scorpine family. Toxin HelaTx1 manifesting the moderate activity against Kv1.1 and Kv1.6 channels belongs to new κ-KTx5 subfamily of potassium channel blockers [5]. One more toxin, hetlaxin, of the scorpion alpha-toxin family possesses high affinity to Kv1.3 potassium channel [3]. The data about coagulopathic properties of this venom are absent.

However, some scorpion venoms cause blood-clotting disorders, but the number of coagulopathic compounds studied to date is quite small. It was reported that venoms of scorpions *Buthotus judaicus*, *Heterometrus spinnifer*, *Parabuthus transvaalicus*, *Androctonus australis*, *Scorpio maurus palmatus*, *Leiurus quinquestriatus habraeus* and *Pandinus imperator* caused an increase of clotting time. In particular, the venoms of *P. imperator* and *P. transvaalicus* species increased the clotting time by 2.5 and 2.3 times, respectively, while other venoms prolonged the time 0.8–2 times [6]. The crude venom of *Buthus tamulus* scorpion caused coagulopathy and might also induce disseminated intravascular coagulopathy (DIC syndrome). The intravenous injection of this scorpion venom at a sublethal dose to dogs and rabbits resulted in a change of the blood coagulation [7].

The investigation of in vitro effects of the venoms from scorpions *Palamneus gravimanus* and *Leiurus quinquestriatus* upon the coagulation of human plasma have shown that the crude venom of *P. gravimanus* has both procoagulant and anti-coagulant properties [8] and the crude venom of *L. quinquestriatus* is very weak anti-coagulant, which shortens the recalcified plasma clotting time by only 5–20% [8]. No fibrinolytic activity was found. Further experiments with fractions of *P. gravimanus* venom, partially purified by DEAE-Sephadex column chromatography, suggest that the procoagulant activity promotes Factor X activation while the anticoagulant fraction interferes with the action of thrombin upon fibrinogen.

It has been shown that a high concentration of *Tityus discrepans* venom in the human blood plasma increases the severity of envenomation symptoms by modifying activated partial thromboplastin time (APTT) and prothrombin time (PT), increasing cytokine level and amylase concentration as well as by inducing hyperglycemia [9]. This scorpion venom was separated into six fractions by gel filtration on a “Protein-Pack 125” column [10]. The investigations of effects on APTT, PT and direct clotting activity, using fresh human plasma and purified fibrinogen as substrates, for crude venom and its fractions showed that the venom and fraction F1 shortened APTT; venom, fraction F6 and fraction F2 prolonged PT. No thrombin-like activity was found with this venom on human plasma or purified fibrinogen [10].

Several fibrin(ogen)olytic enzymes were partially purified from *T. discrepans* venom by different types of liquid chromatography [11]. Two fractions had fibrinolytic, fibrinogenolytic (Aα-chains degradation) and tissue plasminogen activator (t-PA)-like activities; one was only fibrinogenolytic (fast degradation of fibrinogen Aα-chains and slower degradation of Bβ-chains). The fibrino(geno)lytic activity in these fractions was abolished by metalloprotease inhibitors. The other two fractions contained fibrinogenolytic (Aα-chains degradation) and fibronectinolytic activities. Serine protease inhibitors abolished activities in these fractions. None of the fractions degraded fibrinogen γ-chains. Fibrinogen degradation by active fractions was associated with an anticoagulant effect.

Furthermore, two anticoagulant phospholipases A2 (PLA2) were isolated: the imperatoxin (IpTxi)—from the *P. imperator* [12] and the phaiodactylipin—from *Anuroctonus pahiodactylus* [13]. Imperatoxin is a heterodimeric protein with a molecular weight of 14,314 Da. Its molecule consists of a large subunit (104 amino acid residues) that exhibits phospholipase activity, and the small subunit (27 amino acid residues) covalently linked by disulfide bridge to the large subunit. The native heterodimer exhibits hydrolytic activity against phospholipids, although it was originally described as an inhibitor of ryanodine receptor in sarcoplasmic and cardiac reticulums [12]. Phaiodactylipin is a glycosylated dimeric PLA2 with a molecular weight of 19,172 Da. The protein consists of two subunits: the large one comprises 108 amino acid residues and the small subunit—18 residues; the protein structure is stabilized by five disulfide bonds. Phaiodactylipin exerts a hemolytic effect on human erythrocytes and prolongs the blood coagulation time. It should be noted that IpTxi is more effective than pachyodactylipin as anticoagulant; if its concentration in the blood exceeds 10 μg/ml, the clotting time is 30 min [13].

Scorpion venom, along with the above mentioned high molecular weight proteins and PLA2s, also contains polypeptides of smaller molecular mass that affect coagulation. The toxin isolated from the venom of the Chinese scorpion *Buthus martensii Karsch* and called **S**corpion **V**enom **A**ctive **P**olypeptide (SVAP), induced platelet aggregation in rabbit blood in vivo and in vitro. SVAP also caused thrombus formation and a change in levels of thromboxane B2 and 6-keto-prostaglandin F1a in blood plasma. SVAP is the most abundant component of this venom [14]. Recently, a peptide called discreplasminin was isolated from the scorpion *T. discrepans* venom [15]. Discreplasminin had a pI of 8.0 and a molecular weight of about 6 kDa. It strongly inhibited plasmin activity and was suggested to have an anti-fibrinolytic mechanism, similar to that of aprotinin, and to interact with the active site of plasmin.

Previously, we have shown that the fractions obtained by gel filtration of *Heterometrus laoticus* scorpion venom affect the processes of blood coagulation [16] and the structures of low-molecular anticoagulants were established [17]. In the present work, we determined their anticoagulant activity.

## 2. Results

### 2.1. Isolation of Active Compounds

**Figure 1:**
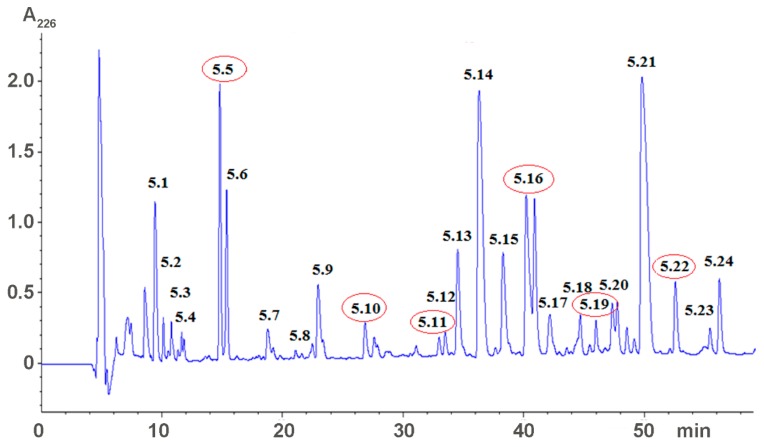
Separation of a low molecular weight fraction by reversed phase HPLC on Eclipse XDB C18 column (9.4 × 250 mm, 5 μm); the gradient of acetonitrile in 0.1% trifluoroacetic acid from 0% to 35% in 70 min. Flow rate 2 mL/min. The presence of polypeptide in the fractions was detected by UV absorbance at 226 nm. Fractions increasing coagulation and bleeding time are indicated by red ellipses.

Earlier, we fractionated the *Heterometrus laoticus* scorpion venom by gel-filtration on Sephadex G-50 column and found that some fractions influenced blood coagulation in vitro and bleeding in mice in vivo [16]. The low-molecular weight fraction V was further separated by reversed phase HPLC (Figure 1) and fractions obtained were tested for effects on blood coagulation and bleeding [18].

**Figure 2:**
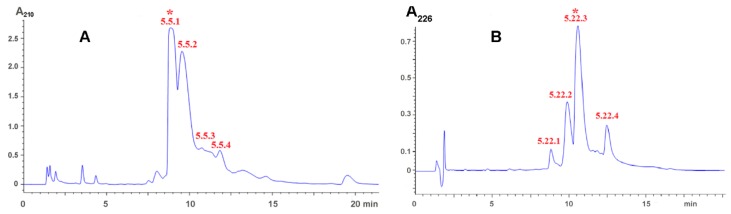
Isolation of active compounds by reversed phase chromatography on Analytical Eclipse XDB C18 column (4.6 × 250 mm, 5 μm) (**A**) Separation of fraction 5.5 in the gradient of acetonitrile in 0.1% trifluoroacetic acid from 0 to 10% in 40 min. Flow rate 1 mL/min; (**B**) Separation of fraction 5.22 in the gradient of acetonitrile in 0.1% trifluoroacetic acid from 15 to 30% in 30 min. Flow rate 1 mL/min.

From 24 fractions obtained, five fractions (5.5, 5.10, 5.11, 5.16, 5.19, and 5.22) significantly increased blood coagulation time in vitro and bleeding time in vivo [18]. The most active fractions, 5.5 and 5.22, were further purified by two more cycles of reversed phase chromatography. Figure 2 shows the first round of purification. The active fractions indicated by asterisks in Figure 2 were re-chromatographed under the same conditions and used for structure determination. The most abundant fraction, 5.21 (Figure 1), was also purified under conditions used for purification of fraction 5.22 and the structure of purified compound was analyzed as well. Analysis of purified substances indicated that fraction 5.5 corresponds to adenosine, fraction 5.21—to dipeptide LeuTrp and fraction 5.22—to dipeptide IleTrp [17]. The large quantities of dipeptides were prepared by peptide synthesis and their anticoagulant activity was analyzed in detail.

### 2.2. Studies of Anticoagulant Activity

#### 2.2.1. Influence on Blood Coagulation In Vitro

To check in vitro anticoagulant activity of synthetic dipeptides in human plasma, we used the standard coagulometric tests including the determination of the activated partial thromboplastin time (APTT), the prothrombin test (PTT), and the thrombin test (TT). In all these tests, we did not find any anticoagulant activity of the dipeptides applied at concentrations up to 100 μM. For dipeptide IleTrp, higher concentrations were used in two tests. It was inactive in TT assay up to 620 μM and in PTT—up to 1.6 mM.

The anticoagulant activity of both dipeptides on the whole mice blood was determined by the Burker’s method [19], and when tested on the whole blood, the synthetic dipeptides showed significant increases in clotting time [17]. To study anticoagulant activity, the synthetic dipeptides were injected into the lateral vein of the mouse tail at a dose of 7.8 nmoles/g, which corresponds to maximal calculated concentration of 110 µM in the peripheral blood.

**Figure 3:**
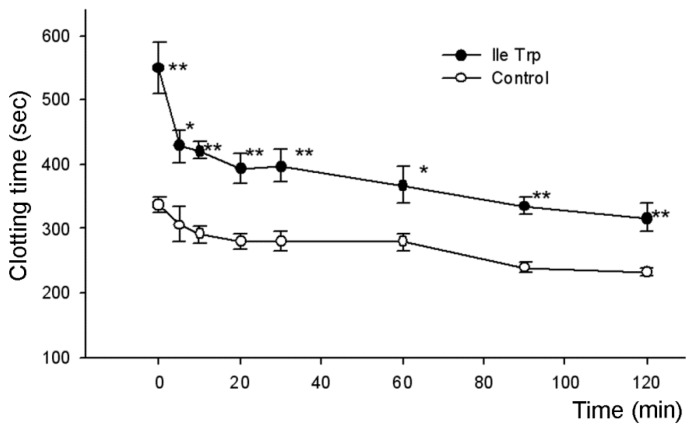
Influence of dipeptide IleTrp on the whole blood coagulation time. The abscissa indicates the time after dipeptide injection. * *p* < 0.05 compared to control; ** *p* < 0.01 compared to control.

The clotting time was determined every 20–30 min during two hours after peptide injection. It was found that the dipeptides at dose of 7.8 nmoles/g significantly prolonged the coagulation time (Table 1). The table also includes data for adenosine and low molecular fraction obtained after the gel-filtration of the crude venom and used for isolation of dipeptides. Adenosine is a well-known inhibitor of platelet aggregation and its injection results in the increase of coagulation time. The statistically significant differences were observed for all tested samples at 20 min after injection, and at one hour after injection only adenosine and IleTrp significantly prolonged clotting time. Although the increase in coagulation time was observed for LeuTrp, it was not statistically significant. Since the most active in this test was dipeptide IleTrp, which significantly increased clotting time even 90 min after injection, its activity was studied in more detail using shorter times after injection (Figure 3): IleTrp strongly prolonged the coagulation time during observation period of two hours. The highest effect was observed immediately after injection and significant differences were registered during the whole observation time.

#### 2.2.2. Influence on Bleeding Time In Vivo

**Figure 4:**
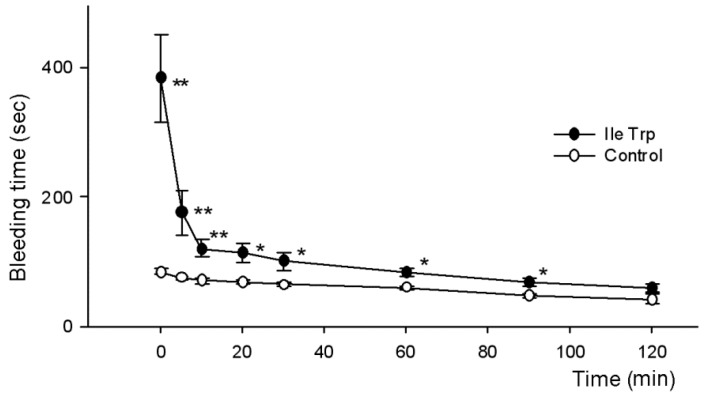
Influence of dipeptide IleTrp on the bleeding time. The abscissa indicates the time after dipeptide injection. * *p* < 0.05 compared to control; ** *p* < 0.01 compared to control.

To determine in vivo anticoagulant activity, solutions of synthetic dipeptides and adenosine were injected in the mice as described above and tail bleeding times were evaluated. As shown in Table 2, tail bleeding times were significantly prolonged by all compounds tested. Similarly to the whole blood clotting test, all samples significantly prolonged bleeding during the first 20 min after injection and only adenosine and IleTrp were active during the first hour. In this test, again the most active was dipeptide IleTrp, which was more active than adenosine 60 min after injection. Although LeuTrp showed a higher effect during the first 20 min, the action of IleTrp was more prolonged. The activity of this dipeptide was studied in more detail using shorter times after injection (Figure 4).

It was found that IleTrp strongly prolonged the bleeding time during the first 10 min. The highest effect was observed immediately after injection and a statistically significant difference between experimental and control mice was maintained up to 90 min.

We have observed no dipeptide effects on plasma coagulation, but have seen strong influence on the whole blood clotting and in vivo bleeding. Basing on these data, we suggested that dipeptides might inhibit platelet aggregation. To check this suggestion, the influence of dipeptides on platelet aggregation was studied.

#### 2.2.3. Influence of Dipeptides on Platelet Aggregation In Vitro

**Figure 5:**
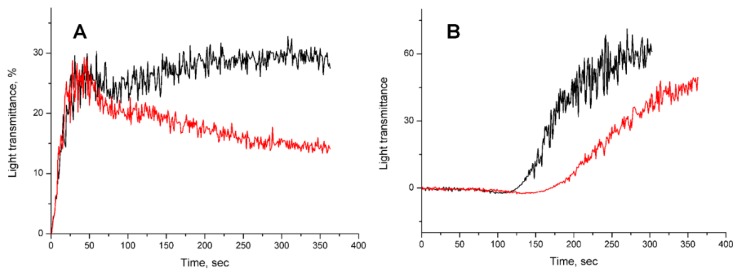
Influence of dipeptide IleTrp on the platelet aggregation. (**A**) ADP induced aggregation. At time zero, ADP was added to platelet rich plasma and light transmittance was registered. The black curve is control (water); the red curve was registered in the presence of IleTrp. (**B**) Collagen induced aggregation. At time zero, collagen was added to platelet rich plasma and light transmittance was measured. The black curve is control (water); the red curve was registered in the presence of IleTrp. Each curve is the mean of two independent measurements.

The effect of dipeptides on platelet aggregation was studied using human platelet rich plasma, which was prepared immediately before use from the blood of healthy donors. The increase in light transmittance upon platelet aggregation was registered and the substance inducing the aggregation by different mechanisms were used. The peptide influence on platelet aggregation induced by addition of ADP, collagen, ristocetin and thrombin was investigated. We have observed practically no effects of IleTrp on aggregation induced by thrombin and ristocetin at peptide concentration up to about 600 μM. The IleTrp also produced no effect, when aggregation was induced by ADP at low concentration and only first aggregation phase was evident. However, at higher ADP concentration inducing two phase aggregation, IleTrp suppressed the secondary phase (Figure 5A), the LeuTrp being much weaker in this assay. When the collagen was used as aggregation inducer, a decrease in initial rate of aggregation was observed in the presence of IleTrp (Figure 5B). The LeuTrp was inactive in this test.

## 3. Discussion

From the venom of scorpion *H. laoticus*, we isolated low molecular weight compounds possessing anticoagulant activity. Determination of their structure showed that one compound was well known anticoagulant adenosine, while the other two were dipeptides LeuTrp and IleTrp [17]. The anticoagulant activity of the isolated substances was studied using standard tests for plasma coagulation (APTT, PTT and TT), whole blood clotting test in vitro and bleeding assay in vivo. In standard tests on plasma, no anticoagulant activity of the dipeptides was observed at concentrations up to 100 μM. The dipeptide IleTrp was inactive in TT at concentration up to 620 μM and in PTT—up to 1.6 mM. However, all three isolated compounds substantially prolonged clotting time of whole blood (Table 1). It was found that at all tested times adenosine at a dose of 2.48 mg/kg (9.3 nmole/g) showed a clotting time greater than in the control group. At 20, 30 and 60 min these differences were statistically significant, while at 90 and 120 min, although adenosine increased clotting time compared to the control group, these differences did not yet have statistical significance. Similarly, the dipeptide IleTrp at a dose of 7.8 nmoles/g significantly prolonged clotting time during all observation time of two hours (Figure 3). The dipeptide LeuTrp at the same dose showed a statistically significant increase in clotting time only at 120 min and was the least active among substances tested. In bleeding in vivo assay all three compounds also prolonged bleeding time (Table 2, Figure 4). The increase in time was very similar to that observed for blood clotting. It should be noted that directly after injection, the IleTrp effect on bleeding was much greater than on clotting (a 4.6-fold increase versus 1.6-fold one).

It should be noted that no systemic bleeding or coagulopathies were reported for *H. laoticus* envenomation [2], therefore the amount of the venom received by the human victims after scorpion sting in not sufficient to affect hemotsasis strongly. Although it is difficult to estimate the amount of venom injected in the prey, the concentrations of dipeptides used in this study are certainly exceeding those achieved in natural scorpion victims. To obtain reliable results, for individual compounds we used the doses of 2.48 mg/kg which corresponds to 1/5 of LD_50_ (12.4 mg/kg) for the crude venom. Certainly the content of each studied compound in *H. laoticus* venom is much less than 20%, however even their low amounts may be sufficient to induce coagulopathies in small animals.

The absence of anticoagulant activity of dipeptides in the standard coagulometric tests on blood plasma might indicate that both intrinsic and extrinsic coagulation pathways were not influenced by these substances, suggesting that the possible target of peptide intervention may be platelet aggregation.

Several platelet receptors involved in platelet activation are known; these are P2Y1 and P2Y12 receptors, thromboxane A2 receptor, PAR-1 and PAR-4 receptors, as well as collagen GPVI and GPIbα receptors [20]. Inhibition of platelet activation is achieved by blocking these receptors. On the other hand, activation of A_2A_ adenosine receptors by adenosine also results in inhibition of platelet aggregation [21]. Thus, sub-micromolar concentrations of adenosine have an antiaggregatory effect on whole human blood [22]. Adenosine has an extremely short lifetime in blood plasma [23]; however, a high dose of 9.3 nmole/g used in our experiments allowed for the detection of the effect. This dose corresponds to the concentration of 133 µM in peripheral blood. At this dose, the observed effects were quite long, the statistically significant increase in both time of coagulation and bleeding was observed within an hour after adenosine administration (Table 1 and Table 2).

Interestingly, the dipeptide IleTrp at slightly lower dose revealed stronger effects; it showed statistically significant elongation of coagulation time during 90 min after administration, with the tendency to increase in time being seen even after two hours (Table 1). Increased bleeding time was observed for two hours after administration, while during the second hour the differences were not statistically significant (Table 2). The observed effect is the first indication that dipeptide can cause an increase in the blood clotting time in vivo.

It should be noted that the tryptophan-containing dipeptides, including IleTrp and LeuTrp, which have been found in food protein hydrolysates, are inhibitors of angiotensin converting enzyme (ACE) involved in the regulation of blood pressure [24]. The peptide IleTrp is a selective and competitive inhibitor of the C-terminal domain of the enzyme possessing a selectivity coefficient of 40 compared to the N-terminal domain [24]. At the same time, increasing evidence suggests that ACE inhibitors (ACE-I) exert antithrombotic effects [25]. Thus it was shown that ACE-I (captopril and lisinopril) enhanced the antiplatelet response to aspirin at concentrations of 15 μM [26]. It was also found that ACE-I exerted an antithrombotic effect in experimental thrombosis in rats [27]. The arterial and venous thrombus weights were reduced after the rats’ treatment with some ACE-I. The same treatments resulted in significant inhibition of the collagen induced platelet aggregation in the whole blood [27]. Pretreatment with ACE-Is resulted in a significant reduction of platelet adhesion to fibrillar collagen. These data are in good agreement with our results on inhibition of collagen induced platelet aggregation by IleTrp (Figure 5).

There is also evidence that the dipeptide IleTrp under trademark BNC210 (Bionomics Limited, Thebarton, Australia) is in the second stage of clinical trials for the treatment of post-traumatic stress disorder [28]. By the mechanism of action, BNC210 is a negative allosteric modulator of nicotinic acetylcholine receptors of the alpha7 type [29]. Given the fact that the aggregation of platelets is substantially inhibited [30] by α-bungarotoxin and methyllycaconitine which are selective antagonists of alpha7 nicotinic cholinergic receptors, one can suggest that the observed anticoagulant effect the dipeptide IleTrp might be mediated by its interaction with this receptor.

Some other molecular mechanisms could be involved in anticoagulant effects of dipeptides. For example, ACE-I, to which IleTrp belongs, have generally been shown to improve the fibrinolytic balance by reducing plasma plasminogen activator inhibitor type 1 (PAI-1) level [31]. The PAI-1 binds to tissue plasminogen activator (t-PA) forming an inactive complex and preventing fibrin breakdown, thus prolonging preservation of thrombus. The reduced PAI-1 level should result in the faster thrombus retraction by t-PA.

The traditional concept of the hemostatic system regulation by a coagulation factor cascade along with platelet activation in recent years has been supplemented by new evidence that the immune system may strongly affect blood coagulation. Under certain conditions, leukocytes can express tissue factor and release proinflammatory and procoagulant molecules [32]. These molecules can influence platelet activation and adhesion as well as activation of the intrinsic and extrinsic coagulation pathways. There is also evidence about multiple interactions between the hemostatic system and innate immunity, and the coagulation and complement cascades. Thus, complement factor 3 (C3) deficiency causes prolonged bleeding, reduced thrombus incidence, thrombus size, fibrin and platelet deposition as well as diminished platelet activation in vitro [33]. Although there are no data about influence of dipeptides on immune system, one cannot exclude their possible action on blood coagulation through effects on immune system.

Interestingly, in in vitro experiments on human platelets, the dipeptides inhibited the secondary phase in aggregation induced by ADP, and IleTrp caused a decrease in initial rate of aggregation induced by collagen (Figure 5). As several mechanisms are involved in the secondary phase in aggregation induced by ADP [34], it is difficult to say which of them is affected by dipeptides. This IleTrp effect on collagen induced aggregation is in good agreement with the literature data. It was shown earlier that in human blood, adenosine A2 receptor agonist CGS 21680 attenuated both in vitro aggregation induced by collagen and flow cytometric markers of platelet activation-aggregation [35]. Moreover, platelet responsiveness to adenosine A2 receptor stimulation was species-dependent: adenosine A2 receptor stimulation inhibited platelet activation by collagen in human, but not canine models. Based on these data, one can suggest that dipeptide effects may be realized though their interaction with adenosine receptors and are stronger in mice than in humans due to species dependence.

However, the above consideration suggests the necessity of further studies to elucidate the exact molecular mechanism of anticoagulant effects of dipeptides.

## 4. Conclusions

From the *H. laoticus* scorpion venom, for the first time we isolated adenosine and two dipeptides LeuTrp and IleTrp possessing anticoagulant activity. The dipeptides did not influence the coagulation time in standard plasma coagulation tests, but, similarly to adenosine, strongly prolonged the bleeding time from mouse tail and in vitro clot formation. The dipeptides inhibited the secondary phase of aggregation induced by ADP, and IleTrp decreased an initial rate of collagen induced platelet aggregation in vitro, which may suggest it interaction with adenosine A_2A_ receptors. One can assume that anticoagulant effects of dipeptides may be realized through the deterioration of platelet function. The ability of short venom peptides to slow down blood coagulation was established for the first time. Further studies are needed to elucidate the precise molecular mechanism of this action and potentially apply it to clinical practice.

## 5. Materials and Methods

### 5.1. Materials

The kits for APTT, PTT, and TT tests as well as thrombin, ADP, ristocetin and collagen were obtained from NPO Renam (Moscow, Russia). Adenosine was from Merck KGaA (Darmstadt, Germany).

#### Scorpions and Scorpion Venom

The scorpions *H. laoticus* were collected in the An Giang province of Vietnam and bred at laboratory of Institute of Applied Materials Science VAST, Ho Chi Minh City. They were fed with crickets and locusts. The scorpions were milked by electrical stimulation, and the venom obtained by this ways was dried over anhydrous CaCl_2_ and kept at −20 °C until use.

### 5.2. Venom Fractionation and Isolation of Low Molecular Weight Compounds

Crude *H. laoticus* venom was first fractionated by gel-filtration Sephadex G50 as described [3]. Five main fractions were obtained. The fraction 5 was further separated by reversed phase HPLC on Eclipse XDB C18 column (9.4 × 250 mm, 5 µm); the gradient of acetonitrile in 0.1% trifluoroacetic acid from 0% to 35% in 70 min. Flow rate 2 mL/min. The presence of polypeptide in the fractions was detected by UV absorbance at 226 nm (Figure 1). The active compounds were further purified by reversed phase chromatography on Analytical Eclipse XDB C18 column (4.6 × 250 mm, 5 µm) Fraction 5.5 was separated in the gradient of acetonitrile in 0.1% trifluoroacetic acid from 0 to 10% in 40 min; fraction 5.21 and 5.22—in the gradient of acetonitrile in 0.1% trifluoroacetic acid from 15 to 30% in 30 min. Flow rate 1 mL/min. The molecular masses of obtained substances were determined by mass-spectrometry on mass-spectrometer LCQ DECA XP+ (Thermo Finnigan, Somerset, NJ, USA).

### 5.3. Characterization of Low Molecular Weight Compounds

The structures of the compounds isolated from fractions 5.5, 5.21 and 5.22 were determined as described [17]. In brief, the molecular mass of the compound from fraction 5.5 was 267.8 Da close to the mass of adenosine (267.2 Da). Both substances co-eluted as a single peak from reversed phase HPLC column and had similar fragmentation mass-spectra. Thus, fraction 5.5 contained adenosine. The compounds from fractions 5.21 and 5.22 had an identical mass of 317.1 Da. The structure of the compound from fraction 5.21 was determined by proton nuclear magnetic resonance. Tandem mass spectrometry analysis of fraction 5.22 showed Leu/IleTrp structure for dipeptide present. As LeuTrp was found in fraction 5.21, fraction 5.22 contained IleTrp dipeptide. The determined structures of dipeptides were confirmed by their chemical synthesis. The peptide synthesis was carried out as described [36].

### 5.4. Mice

Male Swiss albino mice were obtained from the Pasteur Institute of Ho Chi Minh City (Vietnam). The mice were kept at least 2 days prior to the test at Faculty of Pharmacy, Nguyen Tat Thanh University, Ho Chi Minh City. All the appropriate actions were taken to minimize discomfort to mice. World Health Organization’s International Guiding Principles for Biomedical Research Involving Animals were followed during experiments on animals.

### 5.5. Determination of Anticoagulant Activity

To study anticoagulant activity, solutions of synthetic dipeptides and adenosine in 0.9% NaCl were injected into the lateral vein of the mouse tail at a dose of 2.48 mg/kg (injection volume 0.1 mL/10 g of body mouse weight). This dose corresponded to 1/5 of LD50 (12.4 mg/kg) for *H. laoticus* venom at intravenous injection. For dipeptides, 2.48 mg/kg is equal to 7.8 nmoles/g. The average circulating blood volume for mice is 72 mL/kg [37]. The average weight of the mouse used was 20 ± 2 g, the molecular weight of dipeptides—317 Da, and the average blood volume is 1.4 mL; the amount of compounds injected yielded a maximum calculated concentration of 110 µM in the peripheral blood. The mice of the control group received only 0.9% NaCl solution. Each experimental and control group included 6 mice.

#### 5.5.1. Determination of Blood Coagulation Time

The blood coagulation time was determined by modified Burker method [19]. In brief, a drop of blood from the mouse tail was placed on the glass. Every 30 s one tried to tear it away from the glass with the help of an injection needle. The moment when the formed fibrin threads could detach the blood clot from the glass corresponded to the end of the coagulation.

#### 5.5.2. Determination of In Vivo Bleeding Time

Tail bleeding times were measured using the method described by Liu et al. [38]. The distal 5 mm of tail was amputated and the tail (diameter of about 1.5 mm) was immersed in 37 °C solution of 0.9% NaCl. Time to visible cessation of bleeding was recorded.

#### 5.5.3. Platelet Aggregation Measurements

The preparation of platelet rich plasma (PRP) and platelet aggregation measurements using the platelet aggregation analyzer AR2110 (Solar, Minsk, Belarus) were performed essentially as described [39]. In brief, the solution (25 mM) of IleTrp in water (20 µL) was added to 450 µL of PRP and the light transmission was recorded for approximately 30 s. Subsequently, 50 µL of solution containing aggregation inducer was added, and the recording was continued. Thrombin (3 units), ADP at final concentration of 6 μM, ristocetin (1 mg/mL) and collagen at final concentration of 0.02% were used to induce aggregation.

#### 5.5.4. Plasma Coagulation Tests

The plasma coagulation tests were performed using APG2-02 hemostasis analyzer (EMCO, Moscow, Russia) according to the manufacturer protocols.

### 5.6. Statistical Analysis

Significance of differences between experimental and control groups was analyzed by Kruskal-Wallis method and then by Mann-Whitney method using Minitab 15.0 (Minitab Inc., State College, PA, USA) program. The differences were considered significant for *p* values < 0.05. All results are presented as the mean ± SEM (standard error of the mean).

Animal experiments described in this study were approved by the Scientific Council of the Faculty of Pharmacy, Nguyen Tat Thanh University (Protocol No. 1). Protocol was signed by the Chairman of the Council Vice Principal Prof. Nguyen Van Thanh and the Council Secretary Dr. Vo Thi Ngoc My. Date of approval was 19 January 2017.

## Figures and Tables

**Table 1:** Influence of low molecular weight compounds on the mice whole blood coagulation time.

Compound	Time after Injection, min				
	20	30	60	90	120
	Clotting Time, s				
Control	307.7 ± 17.4	290.67 ± 9.58	286.00 ± 6.31	256.3 ± 20.4	229.7 ± 13.2
Fraction 5	422.3 ± 8.4 ^1^	391.7 ± 48.1 ^1^	387.5 ± 35.0	360.2 ± 6.5	358.8 ± 26.6 ^1^
Adenosin	442.5 ± 20.6 ^2^	426.2 ± 25.6 ^2^	366.2 ± 25.0 ^2^	428.3 ± 51.1	296.3 ± 37.4
LeuTrp	401.5 ± 31.2	340.8 ± 29.1	300.3 ± 2.1	460.5 ± 41.7	313.3 ± 24.9 ^1^
IleTrp	556.5 ± 87.2 ^2^	426.2 ± 3.7 ^1^	388.0 ± 46.3 ^1^	367.0 ± 25.5 ^1^	261.8 ± 16.4

^1^ *p* < 0.05 compared to control; ^2^ *p* < 0.01 compared to control.

**Table 2:** Influence of low molecular weight compounds on the bleeding time in mice.

Compound	Time after Injection, min				
	20	30	60	90	120
	Bleeding Time, s				
Control	79.5 ± 13.7	43.33 ± 1.94	45.83 ± 3.95	40.67 ± 5.02	49.67 ± 7.85
Fraction 5	386.2 ± 88.7 ^1^	187.0 ± 64.6 ^1^	86 ± 2.38	119.3 ± 29.2 ^1^	183 ± 80.7
Adenosin	248.2 ± 66.7 ^1^	314 ± 58.6 ^1^	146.7 ± 46.0 ^1^	65 ± 14.5	40.2 ± 10.3
LeuTrp	314.5 ± 85.2 ^1^	84.8 ± 16.7	81.2 ± 15.4	61.8 ± 14.8	68.8 ± 16.4
IleTrp	233.0 ± 30.6 ^2^	179.0 ± 41.4 ^1^	218.7 ± 78.5 ^2^	151.5 ± 57.4	83.8 ± 13.7

^1^ *p* < 0.05 compared to control; ^2^ *p* < 0.01 compared to control.
